# Increased Frequency of Pink Bollworm Resistance to Bt Toxin Cry1Ac in China

**DOI:** 10.1371/journal.pone.0029975

**Published:** 2012-01-04

**Authors:** Peng Wan, Yunxin Huang, Huaiheng Wu, Minsong Huang, Shengbo Cong, Bruce E. Tabashnik, Kongming Wu

**Affiliations:** 1 State Key Laboratory for Biology of Plant Diseases and Insect Pests, Institute of Plant Protection, Chinese Academy of Agricultural Sciences, Beijing, People's Republic of China; 2 Institute of Plant Protection and Soil Science, Hubei Academy of Agricultural Sciences, Wuhan, People's Republic of China; 3 Faculty of Resources and Environmental Science, Hubei University, Wuhan, People's Republic of China; 4 Department of Entomology, University of Arizona, Tucson, Arizona, United States of America; Ghent University, Belgium

## Abstract

Transgenic crops producing insecticidal proteins from *Bacillus thuringiensis* (Bt) kill some key insect pests, but evolution of resistance by pests can reduce their efficacy. The main approach for delaying pest adaptation to Bt crops uses non-Bt host plants as “refuges” to increase survival of susceptible pests. To delay evolution of pest resistance to transgenic cotton producing Bt toxin Cry1Ac, the United States and some other countries have required refuges of non-Bt cotton, while farmers in China have relied on “natural” refuges of non-Bt host plants other than cotton. The “natural” refuge strategy focuses on cotton bollworm (*Helicoverpa armigera*), the primary target of Bt cotton in China that attacks many crops, but it does not apply to another major pest, pink bollworm (*Pectinophora gossypiella*), which feeds almost entirely on cotton in China. Here we report data showing field-evolved resistance to Cry1Ac by pink bollworm in the Yangtze River Valley of China. Laboratory bioassay data from 51 field-derived strains show that the susceptibility to Cry1Ac was significantly lower during 2008 to 2010 than 2005 to 2007. The percentage of field populations yielding one or more survivors at a diagnostic concentration of Cry1Ac increased from 0% in 2005–2007 to 56% in 2008–2010. However, the median survival at the diagnostic concentration was only 1.6% from 2008 to 2010 and failure of Bt cotton to control pink bollworm has not been reported in China. The early detection of resistance reported here may promote proactive countermeasures, such as a switch to transgenic cotton producing toxins distinct from Cry1A toxins, increased planting of non-Bt cotton, and integration of other management tactics together with Bt cotton.

## Introduction

Transgenic crops that produce *Bacillus thuringiensis* (Bt) toxins kill some major insect pests [1]. Transgenic Bt cotton and Bt corn were commercialized in 1996 and grew on more than 58 million hectares worldwide in 2010 [2]. Benefits of these Bt crops can include reduced use of conventional insecticides, regional pest suppression, increased yield, and increased profit [3]–7. The main threat to the long-term efficacy of Bt toxins is evolution of resistance by pests, which entails a genetically based decrease in the susceptibility of pest populations [8]–[11].

Many insects have been selected for resistance to Bt toxins in the laboratory, and some populations of at least eight species of crop pests have evolved some degree of resistance either to Bt sprays outside of the laboratory or to Bt crops in the field [12]–[19]. Although even small decreases in susceptibility can provide the initial evidence of field-evolved resistance, the extent to which such resistance reduces the efficacy of Bt toxins depends on many factors, including the frequency, magnitude, and spatial distribution of resistance [10]. The primary goal of resistance monitoring is to detect field-evolved resistance before control failures occur, so that proactive countermeasures can limit the negative consequences of resistance [10], [20], [21].

The predominant strategy for delaying evolution of pest resistance to Bt crops boosts survival of susceptible insects with “refuges” of host plants that do not produce Bt toxins [8], [9], [20], [21]. Ideally, most of the rare resistant insects emerging from Bt crops will mate with the relatively abundant susceptible insects from nearby refuges. If the dose of Bt toxin ingested by larvae is high enough to kill all or nearly all of the hybrid progeny produced by matings between susceptible and resistant insects, refuges are expected to be especially effective for delaying resistance [9], [20], [21].

Retrospective evaluations of global resistance monitoring data suggest that refuges have delayed pest resistance to Bt crops, especially when the plants have met the “high dose” criterion and refuges have been abundant [10], [11], [21]. In particular, theoretical and empirical analyses imply that refuges have delayed resistance in pink bollworm (*Pectinophora gossypiella*), one of the world's most destructive pests of cotton [21]–[23]. Pink bollworm resistance to Bt cotton has been reported in the field in India, where farmer compliance with the refuge strategy has been low [17], [24]. By contrast, compliance with the refuge strategy is considered a primary reason that pink bollworm susceptibility to Bt cotton did not decrease in the field in Arizona, USA from 1997 to 2005 [21], [22], [25].

In the United States and some other countries, farmers have been required to plant refuges of non-Bt cotton near first-generation Bt cotton that produced Bt toxin Cry1Ac [10], [20]. In the United States and Australia, Bt cotton producing only Cry1Ac is no longer grown and has been replaced largely by Bt cotton that produces two toxins, primarily Cry1Ac and Cry2Ab [10], [26]. Unlike the situation in the United States and Australia, refuges of non-Bt cotton have not been required in China and Bt cotton producing Cry1Ac has not been replaced by two-toxin cotton [27], [28].

In China, Bt cotton producing Cry1Ac was commercialized in 1997 and has been useful against its primary target, the cotton bollworm (*Helicoverpa armigera*), a serious pest of many crops [4], [27], [28]. The lack of a requirement for non-Bt cotton refuges in China is based on the idea that the abundant non-Bt host plants of *H. armigera* other than cotton provide sufficient “natural” refuges to delay resistance in this pest [27]–[29]. Initial evidence of field-evolved resistance of *H. armigera* to Cry1Ac has been detected in China, but field control problems associated with this resistance have not been reported [19], [30], [31]. 

Although pink bollworm is not the primary pest targeted by Bt cotton throughout China, it is a major pest in the Yangtze River Valley of China, where millions of resource-poor farmers plant more than a million hectares of cotton each year, and Bt cotton was introduced in 2000 [32]. Because the pink bollworm feeds almost exclusively on cotton in this region, the “natural” refuge concept does not apply, raising the risk of resistance [27]. In addition, although inherent susceptibility to Cry1Ac is greater for pink bollworm than for *H. armigera*, the concentration of Cry1Ac in Bt cotton varies over time, allowing survival of susceptible larvae of both pests during some of the growing season in China [27], [33], [34]. Unlike the situation in Arizona, where Bt cotton that produced Cry1Ac had virtually 100% efficacy against susceptible pink bollworm larvae [35], field data on larvae exiting from bolls indicate 1 to 11% survival of susceptible pink bollworm on Bt cotton in the Yangtze River Valley during October 2001 and 2002 [33]. Thus, a high dose of Cry1Ac is not maintained against pink bollworm in this region, which further increases the risk of resistance [9], [20], [21], [27].

Here we report data from the Yangtze River Valley on adoption of Bt cotton for 11 years (2000–2010) and resistance monitoring of pink bollworm for six years (2005–2010). These data show that Bt cotton use increased steadily in this region and susceptibility of pink bollworm to Cry1Ac decreased significantly in 2008–2010 compared with 2005–2007.

## Results

### Planting of Bt Cotton

**Figure 1:**
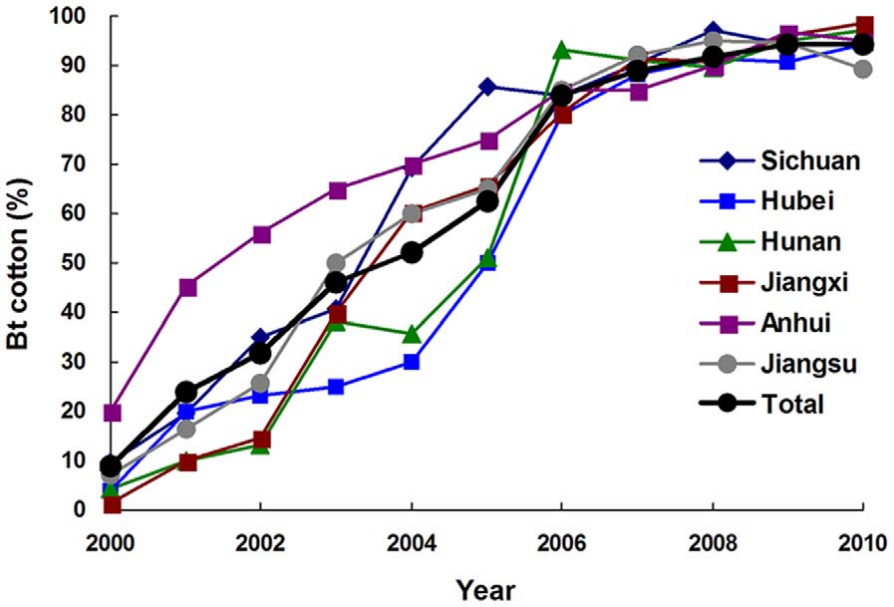
Percentage of all cotton hectares accounted for by Bt cotton in the Yangtze River Valley, 2000–2010.

The percentage of cotton planted with Bt cotton in six provinces of the Yangtze River Valley increased from 9% in 2000 to 52% in 2004, 84% in 2006, 92% in 2008, and 94% in 2009 and 2010 (Fig. 1 and Table S1).

### Resistance to Cry1Ac

**Figure 2:**
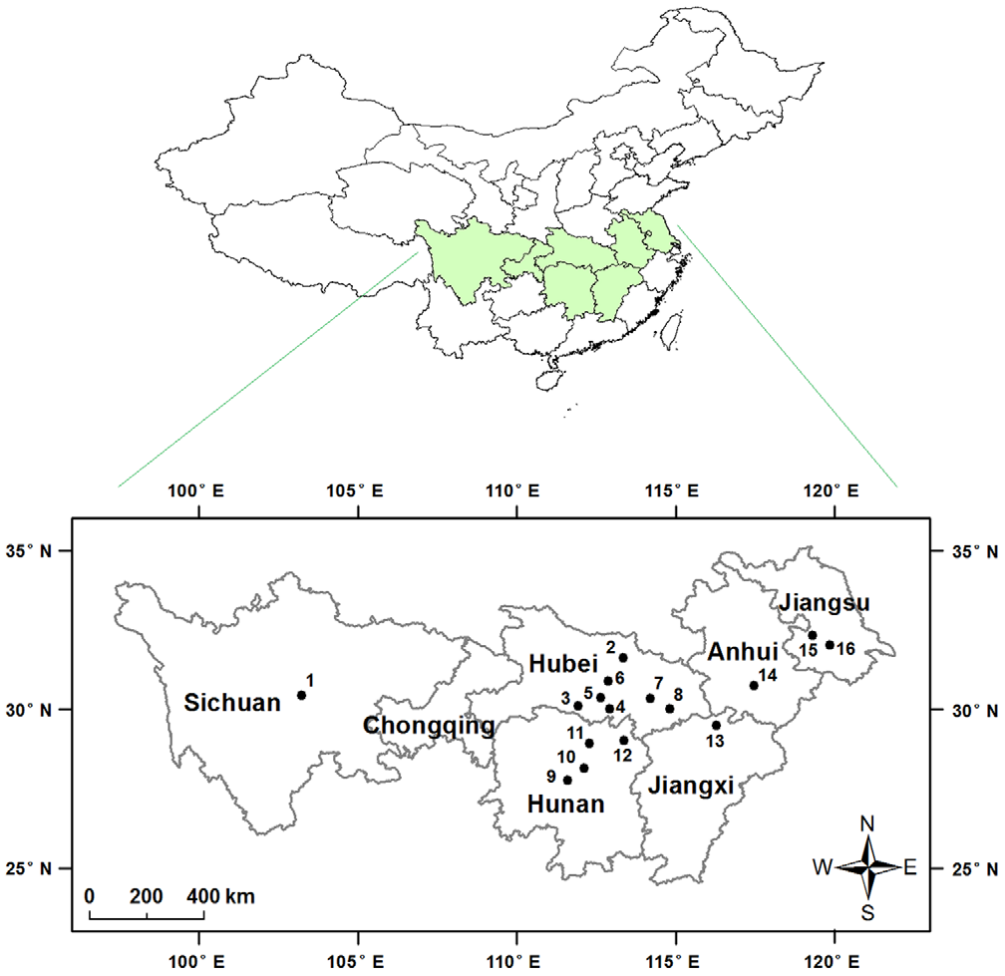
Pink bollworm resistance monitoring field sites in the Yangtze River Valley.

**Figure 3:**
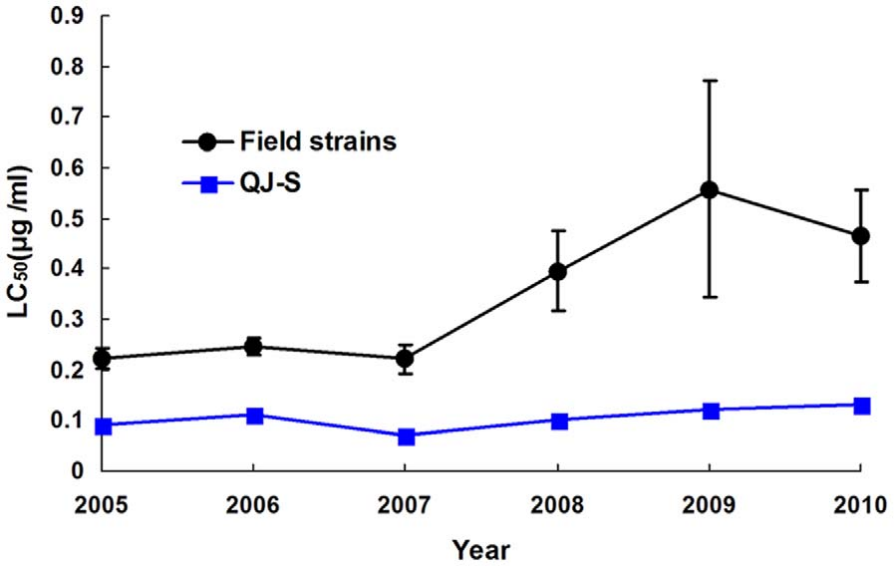
Concentration of Bt toxin Cry1Ac killing 50% of pink bollworm larvae (LC_50_). Field strains were collected from the resistance monitoring sites in the Yangtze River Valley during 2005–2010. The bars represent the standard error of the mean LC_50_ for the field-derived strains tested in a given year (n = 4 to 13 strains per year). QJ-S is a susceptible lab strain that was tested each year as an internal standard.

Susceptibility to Cry1Ac of pink bollworm from the Yangtze River Valley was significantly lower in 2008 to 2010 compared with 2005 to 2007, based on both the concentration killing 50% of larvae (LC_50_) and survival at a diagnostic concentration (9 µg Cry1Ac per ml diet) (Tables 1 and 2, Figs. 2 and 3). The mean LC_50_ (in µg Cry1Ac per ml diet) for Cry1Ac was twice as high in 2008–2010 (0.47) as in 2005–2007 (0.24) (t-test, df = 49, t = 3.0, P = 0.005).

**Table 1:** Responses to Bt toxin Cry1Ac of pink bollworm larvae from the Yangtze River Valley from 2005 to 2007.

Year	Population	Map ref.^a^	Slope	LC_50_	95% fiducial limits			Survival (%)^c^
				µg /ml	Lower	Upper	RR^b^	
2005	Qianjiang	5	1.6	0.20	0.05	0.39	2.2	0.0
	Tianmen	6	1.6	0.28	0.19	0.38	3.1	0.0
	Wuhan	7	1.5	0.19	0.05	0.37	2.1	0.0
	Huanggang	8	1.9	0.22	0.09	0.37	2.4	0.0
	QJ-S		1.2	0.09	0.04	0.17	1.0	0.0
2006	Jianyang	1	2.7	0.29	0.22	0.35	2.6	0.0
	Suizhou	2	2.0	0.12	0.08	0.15	1.1	0.0
	Jingzhou	3	1.7	0.26	0.20	0.33	2.4	0.0
	Qianjiang	5	1.6	0.27	0.11	0.47	2.5	0.0
	Tianmen	6	0.7	0.23	0.11	0.39	2.1	0.0
	Wuhan	7	2.5	0.27	0.21	0.33	2.5	0.0
	Huanggang	8	1.5	0.27	0.11	0.48	2.4	0.0
	Changde	10	2.3	0.20	0.06	0.32	1.8	0.0
	Anxiang	11	1.3	0.25	0.17	0.33	2.3	0.0
	Pengze	13	2.5	0.26	0.05	0.42	2.3	0.0
	Anqing	14	1.5	0.38	0.27	0.51	3.5	0.0
	Nanjing	15	2.4	0.21	0.07	0.34	1.9	0.0
	Jurong	16	2.0	0.20	0.03	0.35	1.8	0.0
	QJ-S		1.1	0.11	0.06	0.16	1.0	0.0
2007	Jianyang	1	1.6	0.20	0.16	0.25	2.9	0.0
	Qianjiang	5	1.6	0.21	0.09	0.36	3.1	0.0
	Wuhan	7	0.8	0.26	0.16	0.51	3.7	0.0
	Huanggang	8	1.7	0.12	0.09	0.15	1.7	0.0
	Anxiang	11	1.1	0.16	0.12	0.22	2.3	0.0
	Anqing	14	1.1	0.35	0.17	2.01	4.9	0.0
	Jurong	16	1.6	0.25	0.19	0.31	3.6	0.0
	QJ-S		1.1	0.07	0.05	0.09	1.0	0.0

a See Fig. 2 for locations indicated by map reference numbers.

b RR (Resistance ratio)  =  LC_50_ of strain/LC_50_ of the susceptible lab strain QJ-S.

c Adjusted survival at a diagnostic concentration of Cry1Ac (see Methods).

**Table 2:** Responses to Bt toxin Cry1Ac of pink bollworm larvae from the Yangtze River Valley from 2008 to 2010.

Year	Population	Map ref.^a^	Slope	LC_50_	95% fiducial limits			Survival (%)^c^
				µg /ml	Lower	Upper	RR^b^	
2008	Jianyang	1	1.4	0.25	0.19	0.32	2.5	0.0
	Qianjiang	5	1.5	0.33	0.18	0.50	3.3	0.0
	Tianmen	6	1.1	0.21	0.04	0.44	2.1	1.6
	Wuhan	7	1.3	0.42	0.20	0.72	4.2	1.8
	Huanggang	8	1.6	0.39	0.31	0.49	3.9	0.0
	Changde	10	1.5	0.92	0.67	1.21	9.1	3.6
	Anxiang	11	1.9	0.38	0.19	0.62	3.8	0.0
	Anqing	14	1.3	0.26	0.19	0.33	2.6	0.0
	QJ-S		3.0	0.10	0.06	0.14	1.0	0.0
2009	Jianyang	1	0.9	0.28	0.15	0.44	2.4	5.4
	Qianjiang	5	1.2	1.73	0.43	8.35	14.6	3.6
	Tianmen	6	1.2	1.24	0.04	9.82	10.5	1.8
	Huanggang	8	1.5	0.12	0.07	0.17	1.0	0.0
	Changde	10	1.7	0.17	0.00	0.44	1.4	0.0
	Anxiang	11	1.6	0.54	0.38	0.71	4.6	0.0
	Pengze	13	0.8	0.12	0.04	0.21	1.0	8.6
	Anqing	14	1.1	0.26	0.00	0.70	2.2	1.6
	QJ-S		1.4	0.12	0.06	0.19	1.0	0.0
2010	Jianyang	1	1.4	0.33	0.25	0.41	2.6	0.0
	Jianli	4	1.5	1.30	0.88	1.84	10.3	4.1
	Qianjiang	5	2.3	0.17	0.08	0.26	1.4	0.0
	Tianmen	6	1.2	0.29	0.22	0.37	2.3	1.8
	Huanggang	8	1.1	0.38	0.26	0.52	3.0	1.6
	Taoyuan	9	1.0	0.27	0.10	0.52	2.1	0.0
	Changde	10	1.8	0.52	0.40	0.65	4.1	0.0
	Anxiang	11	1.7	0.33	0.17	0.53	2.6	1.1
	Yueyang	12	1.6	0.53	0.38	0.70	4.2	3.3
	Pengze	13	1.4	0.44	0.34	0.55	3.4	5.4
	Anqing	14	1.6	0.56	0.44	0.71	4.5	5.6
	QJ-S		2.0	0.13	0.12	0.18	1.0	0.0

a See Fig. 2 for locations indicated by map reference numbers.

b RR (Resistance ratio)  =  LC_50_ of strain/LC_50_ of the susceptible lab strain QJ-S.

c Adjusted survival at a diagnostic concentration of Cry1Ac (see Methods).

The percentage of populations with one or more larvae surviving at the diagnostic concentration increased from 0% (n = 24 populations) during 2005–2007 to 56% (15 of 27 populations) during 2008–2010 (Fisher's exact test, P<0.0001). In addition, the median percentage survival at the diagnostic concentration increased from 0% in 2005–2007 to 1.6% in 2008–2010 (Mann-Whitney U-test, U = 504, P<0.001). We found survivors at the diagnostic concentration in three consecutive years (2008–2010) in the Tianmen population from Hubei province and in two consecutive years (2009–2010) in the Pengze population from Jiangxi province and in the Anqing population from Anhui province (Table 2). Survival at the diagnostic concentration and the LC_50_ value were positively correlated across all bioassays (Spearman's r_s_ = 0.50, df = 55, P<0.0001).

The simplest explanation for the observed increases over time in LC_50_ values and survival at the diagnostic concentration is that the frequency of resistance to Cry1Ac increased in the field populations of pink bollworm tested. An alternative hypothesis is that conditions in the laboratory changed over time in a way that increased survival in bioassays. For example, this could have happened if the Cry1Ac used in bioassays was less potent in 2008–2010 than in 2005–2007. However, this alternative hypothesis is not supported by the data for the susceptible lab strain QJ-S, which show no significant increase from 2005–2007 to 2008–2010 in either LC_50_ (0.09 for 2005–2007 versus 0.12 for 2008–2010; t-test, df = 4, t = 1.8, P = 0.14) or survival at the diagnostic concentration (0% in all years).

Nonetheless, because of the numerically higher LC_50_ for QJ-S in 2008–2010 relative to 2005–2007, we also used the conservative approach of comparing resistance ratios over time. This approach accounts for any increases in the LC_50_ of QJ-S over time, because we calculated resistance ratios as the LC_50_ value of a field population divided by the LC_50_ value for QJ-S tested in the same year. The resistance ratio of field populations was significantly higher during 2008–2010 (4.1) compared with 2005–2007 (2.5) (t-test, t = 2.2, df = 49, P = 0.03). In addition, the maximum resistance ratio was only 4.9 during 2005–2007 (2007: Anqing), whereas three populations tested during 2008–2010 had resistance ratios >10 (2009: Qianjiang  = 14.6 and Tianmen  = 10.5; 2010: Jianli  = 10.3; Tables 1 and 2). Thus, analysis of LC_50_ values, survival at a diagnostic concentration, and resistance ratios support the conclusion of field-evolved resistance to Cry1Ac by pink bollworm in the Yangtze River Valley.

**Figure 4:**
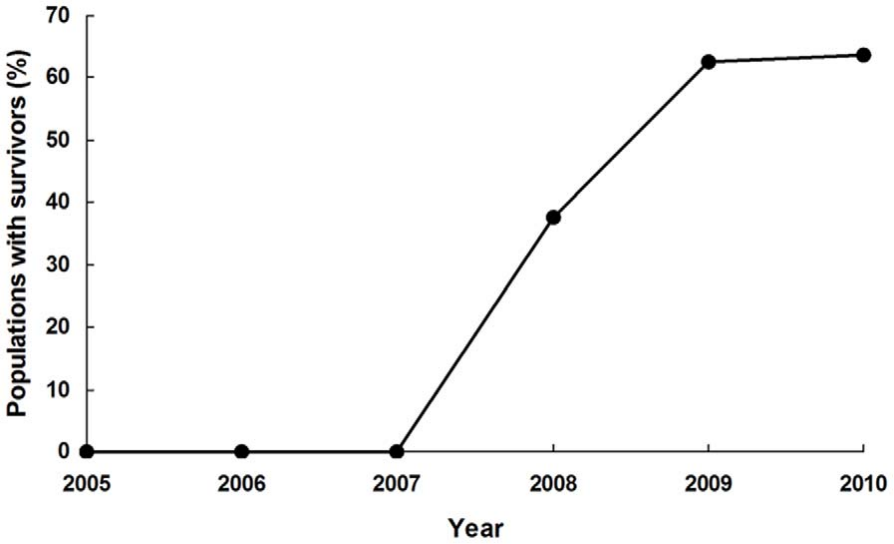
Percentage of pink bollworm field populations with survivors at the diagnostic concentration (9 µg Cry1Ac per ml diet). The number of field populations tested in the Yangtze River Valley was 4 in 2005, 13 in 2006, 7 in 2007, 8 in 2008, 8 in 2009, and 11 in 2010 (see Tables 1 and 2).

Although susceptibility to Cry1Ac was lower in 2008–2010 than in 2005–2007, susceptibility to Cry1Ac did not decrease from 2009 to 2010. The mean LC_50_ value was slightly lower in 2010 (0.47) than in 2009 (0.56), but this difference was not significant (t-test, df = 17, t = 0.44, P = 0.67) (Fig. 3). The percentage of field populations with one or more survivors at the diagnostic concentration was nearly identical in 2009 (62%) and 2010 (64%) (Fisher's exact test, P = 1.0) (Fig. 4). Therefore, resistance to Cry1Ac did not increase from 2009 to 2010, despite Bt cotton accounting for 94% of the cotton planted in the Yangtze River Valley in both years (Table S1).

### Survival on Bt Cotton Bolls

Larval survival on Bt cotton bolls was significantly higher for the lab-selected resistant strain YZP06-R (1.5%) than for the susceptible lab strain QJ-S (0.0%) (t-test, t = 3.2, df = 4, P = 0.032, Table 3). On non-Bt cotton bolls, however, larval survival did not differ significantly between the resistant strain (56.2%) and the susceptible strain (48.8%) (t-test, t = 1.6, df = 4, P = 0.19, Table 3). We also calculated relative survival as larval survival on Bt cotton divided by larval survival on non-Bt cotton, which was significantly higher for the resistant strain (2.8%) than for the susceptible strain (0.0%) (t-test, t = 3.0, df = 4, P = 0.039). The number of entry holes per boll did not differ significantly between strains for Bt bolls, non-Bt bolls, or all bolls pooled (t-tests, P>0.07 in each case).

**Table 3:** Survival on bolls of Bt and non-Bt cotton of pink bollworm larvae from a susceptible strain (QJ-S) and a lab-selected resistant strain (YZP06-R).

Strain	Cotton type	Number of bolls	Entry holes per boll	Survival (%) a	Relative survival (%)^b^
YZP06-R	Bt	150	3.3 (0.5)	2.1 (0.5)	2.8 (0.9)
QJ-S	Bt	150	4.3 (0.4)	0.0 (0.0)	0.0 (0.0)
YZP06-R	Non-Bt	150	3.1 (0.3)	56.2 (1.4)	
QJ-S	Non-Bt	150	3.6 (0.3)	48.8 (4.5)	

Values are means with their standard errors in parentheses.

a Larvae surviving per boll divided by entry holes per boll multiplied by 100%.

b Survival on Bt cotton divided by survival on non-Bt cotton.

Results from diet bioassays performed simultaneously with the boll bioassays showed that the LC_50_ value (µg Cry1Ac per ml diet with 95% fiducial limits) was 7.25 (5.6–11) for the resistant strain and 0.11 (0.07–0.14) for the susceptible strain, which yields a resistance ratio of 66. Survival at the diagnostic concentration was 40% for the resistant strain and 0% for the susceptible strain (n = 72 larvae for each strain).

## Discussion

The results reported here show significantly decreased pink bollworm susceptibility to Bt toxin Cry1Ac in the Yangtze River Valley of China during 2008 to 2010 compared with 2005 to 2007, based on LC_50_ values, survival at a diagnostic concentration, and resistance ratios (Tables 1 and 2, Figs. 3 and 4). The first evidence of pink bollworm resistance to Bt cotton was detected in 2008 (Table 2, Figs. 3 and 4), eight years after Bt cotton was introduced in the Yangtze River Valley. However, non-Bt cotton accounted for more than 37% of the total cotton planted in this region until 2006, when the non-Bt cotton percentage dropped to 16% (Table S1). Thus, the first evidence of field-evolved resistance to Cry1Ac in the region was detected only two years after Bt cotton exceeded 80% of the total area of cotton planted.

Although the results here show significant decreases in pink bollworm susceptibility to Cry1Ac, data showing failure of Bt cotton producing Cry1Ac to control pink bollworm have not been reported from the Yangtze River Valley. However, our results do show that survival on bolls of Bt cotton was significantly higher for a lab-selected resistant strain derived from the Yangtze River Valley in 2006 (YZP06-R) than for a susceptible strain (Table 3). For this resistant strain, larval survival on Bt cotton bolls relative to non-Bt cotton bolls was only 2.8% (Table 3), but this could underestimate the potential for increased survival on Bt cotton in the field for two reasons. First, the bolls tested in our bioassays were collected from GK19 Bt cotton plants in the field during August, when this type of cotton is highly effective against pink bollworm [33]. Mean survival in the field on GK19 was only 1.3% (range  = 0 to 2.6%) in August 2001 and 2002, compared with 11% (range  = 10 to 11%) in October 2001 and 2002 [33]. Moreover, the YZP06-R strain had only 40% survival at a diagnostic concentration of Cry1Ac, which implies that 60% of the larvae tested from this strain had little or no resistance to Cry1Ac. We expect that at the end of the season, survival on Bt cotton bolls for a field population with a higher frequency of resistance would be higher than the survival we observed for the YZP06-R strain on Bt cotton bolls collected from the field in August.

The finding that survival of pink bollworm larvae on Bt cotton in the Yangtze River Valley was as high as 11% during October 2001 and 2002 [33], six and seven years before field-evolved resistance was first detected in 2008, implies that Bt cotton did not kill all or nearly all susceptible larvae. Thus, we infer that even small decreases in susceptibility to Cry1Ac could reduce the efficacy of Bt cotton in the field. Based on the field data described above and our bioassay results, we hypothesize that the magnitude of resistance documented here reduces the efficacy of Cry1Ac-producing Bt cotton against pink bollworm in the field, at least during some part of the growing season. On the other hand, the median percentage survival at a diagnostic concentration of Cry1Ac was only 1.6% for 2008 to 2010 (Table 2), which indicates that the frequency of resistance was too low to cause major field control problems during those three years.

Comparison of pink bollworm resistance to Bt cotton producing Cry1Ac across China, India, and the United States suggests that the refuge strategy has helped to delay resistance. The two key conditions favoring the effectiveness of the refuge strategy are sufficient refuges of non-Bt host plants and a toxin concentration in Bt plants that is high enough to kill all or nearly all hybrid progeny, which is the so-called “high dose” criterion [9]–[11], [21]. In China and India, these two key conditions have not been met, and pink bollworm has evolved resistance to Cry1Ac. In China, non-Bt cotton refuges have not been required. In India, farmers apparently have not complied with the refuge requirement [24]. In both China and India, Bt cotton producing Cry1Ac has not met the “high dose” standard against pink bollworm. Field data from these two countries show substantial survival of susceptible pink bollworm larvae on approved varieties of Bt cotton that produce Cry1Ac [33], [35], [36], which implies a failure to meet the high dose criterion. In China, most Bt cotton planted has been a type called GK19 that was developed by the Biotechnology Research Institute of the Chinese Academy of Agricultural Sciences and contains a chimeric *cry1Ac/cry1Ab* gene [34]. In field trials in the Yangtze River Valley, the mean number of pink bollworm larvae per 100 bolls in October 2001 and 2002 was about five times higher for GK19 (10.6) than for BG1560 (2.2), a type of Bt cotton from Monsanto that contains a *cry1Ac* gene [33]. In Arizona, however, both key conditions of the refuge strategy were met, and pink bollworm susceptibility to Cry1Ac did not decrease, despite a relatively high initial frequency of resistance [7], [22], [37], [38].

In India, pink bollworm resistance to Cry1Ac was detected first in a single field population sampled in 2008 from the state of Gujarat that had a resistance ratio of 42 to 47 in lab diet bioassays [17] similar to the diet bioassays used here. In response to this finding, no major changes in resistance management were implemented, and unusually high pink bollworm abundance occurred on Bt cotton producing Cry1Ac in four districts of Gujarat during 2009 [39]. By contrast, the highest Cry1Ac resistance ratio for pink bollworm in China was only 14.6 (Qianjiang 2009, Table 2) and resistance to Cry1Ac did not increase in the Yangtze River Valley from 2009 to 2010. Nonetheless, the significantly increased resistance detected in 2008 to 2010 compared with 2005 to 2007 suggests that countermeasures should be considered now. The events in India and the resistance monitoring data reported here provide a warning that may be early enough to spur proactive measures to limit any negative consequences of pink bollworm resistance to Cry1Ac in China.

One option to counter resistance is to switch to Bt cotton that produces Cry2Ab and Cry1Ac, which is effective against pink bollworm with high levels of lab-selected resistance to Cry1Ac [40]. This option is one of the most feasible, because such two-toxin Bt cotton is already approved for small-scale trials in China. This option would also be useful for countering *H. armigera* resistance to Cry1Ac [19]. However, for long-term sustainable control, cotton with two or more toxins other than Cry1Ac would be better for countering resistance to Cry1Ac [19], [41]. A second option is to increase planting of non-Bt cotton. A third approach is to integrate other control tactics with Bt cotton for management of pink bollworm. For example, sterile moth releases and other tactics have been used in combination with Bt cotton to suppress pink bollworm in the United States [7]. This approach has been implemented in Arizona since 2006 and pink bollworm has remained susceptible to Cry1Ac, even when the percentage of cotton planted with Bt cotton exceeded 96% statewide [7]. The success of this strategy in Arizona suggests that it could be considered, at least as a supplemental measure, for managing pink bollworm resistance to Bt cotton in China.

## Materials and Methods

### Area Planted to Bt Cotton and Non-Bt Cotton

For each year from 2000 to 2010, we obtained for each of six provinces of the Yangtze River Valley (Sichuan, Hubei, Hunan, Jiangxi, Anhui and Jiangsu) the area planted to cotton from the China Agriculture Yearbook [32] and the area planted to Bt cotton from the Ministry of Agriculture of China. For each year, we calculated the percentage of cotton planted with Bt cotton as the area planted with Bt cotton divided by the total area planted to cotton, multiplied by 100%.

### Insect Rearing

We reared larvae on wheat germ diet [42], [43] at 30°C, 60% RH and 24 h light. The adults of each field strain were placed in cages (50 by 50 by 50 cm) and provided with 5% honey solution. The top of each cage was covered with white gauze for oviposition. Egg gauzes were harvested daily during the entire oviposition period and placed in plastic bags until egg hatching.

### Insect Strains

To monitor susceptibility of field populations, we collected 50 to 300 *P. gossypiella* larvae per site at 4 to 13 sites per year from cotton fields in the Yangtze River Valley during July to November 2005 to 2010 (Fig. 2). The resistance monitoring was limited to Hubei Province in 2005 and expanded to the entire Yangtze River Valley in 2006–2010. The collection sites varied among years because pink bollworm abundance varied and we could conduct bioassays only in cases where the collection from a site yielded enough individuals to initiate a field-derived strain.

In China, farmers pick cotton by hand from their small farms. Cotton companies buy the harvested raw cotton from farmers near their fields. When raw cotton accumulates at purchasing sites, the high temperature inside cotton bolls causes pink bollworm larvae to exit from the bolls. Therefore, it is much easier to collect larvae from purchasing sites than directly from cotton fields. For 50 of the 51 collections from field sites used to start strains for bioassays, we obtained larvae from purchasing sites. The bolls at each of these 50 sites were a mixture of Bt and non-Bt cotton that reflected the proportions of Bt and non-Bt cotton harvested at each site. Thus, the larvae collected were representative of the populations at each site. In the exceptional case, we obtained larvae from non-Bt cotton bolls from the Wuhan site in 2005. No permits were required because all collections were made in China under the auspices of the Chinese Academy of Agricultural Sciences. We mass-reared the field-collected larvae from each site separately on diet in the laboratory and tested their F1 progeny using diet bioassays as described below. We tested 51 strains that were derived from the field during 2005 to 2010.

As an internal control, we also tested a susceptible strain (QJ-S) each year in conjunction with diet bioassays of the 51 field-derived strains (see below). The QJ-S strain was started with insects collected from Qianjiang, Hubei, China in 2004 and reared in the laboratory without exposure to toxins. In addition, the QJ-S strain was used as an internal control in diet and boll bioassays with a lab-selected resistant strain (YZP06-R) (see below).

### Diet Bioassays

Susceptibility to Cry1Ac was determined using diet incorporation bioassays with MVPII (Dow AgroSciences, Indianapolis, USA), which is a formulation containing protoxin that is similar to Cry1Ac [42], [43]. We added sterilized distilled water to produce a stock dilution of MVPII. The stock dilution was added to liquid wheat germ diet in amounts necessary to create final concentrations of 0 (control), 0.05, 0.1, 0.25, 0.5, 1, 2.5, 5 and 9 µg Cry1Ac per ml solution in 2005; and 0, 0.1, 0.2, 0.4, 0.8, 1.6, 3.2 and 9 µg Cry1Ac per ml from 2006 to 2010. Diet was made in 1 liter batches of each concentration, cooled, shredded into pieces (ca. 2 by 1 by 1 cm) and dispensed into 24-well culture plates (Haimeng Shengbang Laboratory Equipment Co., China)with 3 g diet per well. Neonates were placed individually in each well. For testing of the 51 field-derived strains and concomitant tests of QJ-S, we assigned larvae to replicates consisting of three or four bioassay trays per replicate for each concentration (96 larvae per concentration in 2005, 2006, 2007 and 2010; 72 larvae per concentration in 2008 and 2009). Bioassay trays were placed in a growth chamber and incubated in darkness at 29±1°C. After 21 days, live fourth instars and pupae were scored as survivors [42], [43]. Based on bioassays done during 2004, we estimated that the LC_99_ of the susceptible QJ-S strain was 8.4 µg Cry1Ac per ml diet (LC_50_ = 0.17, with 95% fiducial limits of 0.13 and 0.22; slope of 1.37 and SE  = 0.12). Here we used a diagnostic concentration of 9 µg Cry1Ac per ml diet, which is equivalent to the LC_99.86_ of the QJ-S strain in 2004 and is similar to the 10 µg Cry1Ac per ml diet used as a diagnostic concentration for pink bollworm from Arizona [37].

### Laboratory Selection for Resistance

We created the lab-selected, resistant YZP06-R strain as follows: We started with larvae collected in 2006 from four sites in the Yangtze River Valley (Qianjiang, Wuhan, Tianmen and Huanggang). The F1 progeny derived from these four sites were tested with diet bioassays and all 93 survivors at 0.4, 0.8 and 1.6 μg Cry1Ac per ml of diet were pooled to generate YZP06-R. After this pooling, the F1 progeny of YZP06-R were reared without exposure to Cry1Ac. The F2-F6 progeny were reared on diet with 1 μg of Cry1A per ml of diet and the F7-F10 progeny were reared on diet with 2 μg of Cry1Ac per ml of diet.

To determine responses to Cry1Ac, larvae from QJ-S and the F11 generation of YZP06-R were tested with diet bioassays in August 2009, using the procedures described above. Each strain was tested against seven concentrations of Cry1Ac including a control with no toxin added to diet, with 24 larvae tested at each concentration in each of three replicates. The total sample size for each strain was 504 larvae.

### Boll Bioassays

We tested the susceptible QJ-S strain and the F11 generation of the resistant YZP06-R strain in laboratory bioassays with bolls collected from the field in 2009 from Bt and non-Bt cotton plants. We collected bolls from fields of Bt cotton (GK19) and non-Bt cotton (Simian3) in Wuhan, Hubei province. The Bt toxin produced by GK19 is encoded by a chimeric *cry1Ac/cry1Ab* gene [34]. On 15 August 2009, we tagged white flowers on cotton plants. From 1700 to 1900 h on 25 August 2009, we collected 600 bolls (approximately 2 cm in diameter) from the locations of the tagged flowers. We collected a total of 600 bolls; 6 bolls per plant from 50 Bt cotton plants and 50 non-Bt cotton plants. Bolls were placed in cylindrical polyethylene containers (11 cm in diameter by 8 cm high) with 5 bolls per container and transported for about one hour to the lab.

We started the boll bioassays the following morning (0800 to 1000 h) by using a fine brush to transfer 10 neonates to each of the five bolls in each container. In the lab, both before and during boll bioassays, the containers were held in chambers at 29°C, 14L:10D, and 50–70% humidity. Three days after infestation, we counted the entry holes in each boll with the aid of microscopes. Fourteen days after infestation, all bolls were checked for emergence holes. Twenty-one days after infestation, we dissected all bolls and counted the live larvae. The number of survivors was calculated as the number of emergence holes plus live larvae inside bolls. For each insect strain in each replicate, we used 50 bolls of Bt cotton and 50 bolls of non-Bt cotton. We replicated the boll bioassays three times, testing a total of 6000 neonates, 1500 per strain on Bt cotton and 1500 per strain on non-Bt cotton.

### Data Analysis

We analyzed diet bioassay data with probit regression using POLO [44] to determine LC_50_ values and their 95% fiducial limits, and slopes of the concentration- mortality lines. We calculated the resistance ratio as the LC_50_ for a strain divided by the LC_50_ for the susceptible QJ-S strain tested in the same year. All values reported for survival at the diagnostic concentration of 9 µg Cry1Ac per ml diet were adjusted for control mortality. These values were calculated as survival at this concentration divided by survival on control diet, which is equivalent to correcting for control mortality with Abbott's method and calculating adjusted survival as 100% minus adjusted mortality [37].

In the early stages of resistance evolution, stochastic factors can affect the frequency of resistance and the results of resistance monitoring, particularly with relatively small sample sizes over short time intervals when the frequency of resistance is close to the limit of detection. To minimize such effects, we focused primarily on comparisons between 2005–2007 and 2008–2010, which increased the time interval and the sample size for comparisons. We used Fisher's exact test to determine if the proportion of populations with one or more survivors at the diagnostic concentration differed between 2005–2007 and 2008–2010. We used the Mann-Whitney U-test to determine if percentage survival at the diagnostic concentration differed between 2005–2007 and 2008–2010. We used Spearman's rank correlation to test the association between LC_50_ and survival at the diagnostic concentration. Because the sampling method for the strain derived from Wuhan during 2005 differed from the method used for all other samples, we performed all statistical tests with and without the Wuhan 2005 data. The results with and without data from Wuhan 2005 were virtually identical and we report statistical analyses with the Wuhan 2005 data included.

For the boll bioassays, we calculated larval survival in each of the three replicates as the number of survivors divided by the number of entry holes [45]. We also calculated relative survival for each of the three replicates as larval survival on Bt cotton divided by larval survival on non-Bt cotton. We used t-tests to determine if significant differences occurred between the resistant strain (YZP06-R) and the susceptible strain (QJ-S) in larval survival on Bt cotton, larval survival on non-Bt cotton, and relative survival, as well as entry holes per boll on Bt cotton, non-Bt cotton, and all bolls pooled.

## Supporting Information

Table S1 Planting of Bt cotton and non-Bt cotton in the Yangtze River Valley from 2000 to 2010.
(DOC)

## References

[1] Sanahuja G, Banakar R, Twyman R, Capell T, Christou P (2011). *Bacillus thuringiensis*: a century of research, development and commercial applications.. Plant Biotech J.

[2] James C (2010). ISAAA Brief No.39Global Status of Commercialized Biotech/GM Crops: 2010..

[3] Carrière Y, Ellers-Kirk C, Sisterson M, Antilla L, Whitlow M (2003). Long-term regional suppression of pink bollworm by *Bacillus thuringiensis* cotton.. Proc Natl Acad Sci U S A.

[4] Wu KM, Lu YH, Feng HQ, Jiang YY, Zhao JZ (2008). Suppression of cotton bollworm in multiple crops in China in areas with Bt toxin-containing cotton.. Science.

[5] Hutchison WD, Burkness EC, Mitchell PD, Moon RD, Leslie TW (2010). Areawide suppression of European corn borer with Bt maize reaps savings to non-Bt maize growers.. Science.

[6] National Research Council (2010). The impact of genetically engineered crops on farm sustainability in the United States..

[7] Tabashnik BE, Sisterson MS, Ellsworth PC, Dennehy TJ, Antilla L (2010). Suppressing resistance to Bt cotton with sterile insect releases.. Nat Biotechnol.

[8] Tabashnik BE (1994). Evolution of resistance to *Bacillus thuringiensis*.. Annu Rev Entomol.

[9] Gould F (1998). Sustainability of transgenic insecticidal cultivars: integrating pest genetics and ecology.. Annu Rev Entomol.

[10] Tabashnik BE, Van Rensburg JB, Carriere Y (2009). Field-evolved insect resistance to Bt crops: definition, theory, and data.. J Econ Entomol.

[11] Carrière Y, Crowder DW, Tabashnik BE (2010). Evolutionary ecology of insect adaptation to Bt crops.. Evol Appl.

[12] Tabashnik BE, Cushing NL, Finson N, Johnson MW (1990). Field development of resistance to *Bacillus thuringiensis* in diamondback moth (Lepidoptera:Plutellidae).. J Econ Entomol.

[13] Janmaat AF, Myers JH (2003). Rapid evolution and the cost of resistance to *Bacillus thuringiensis* in greenhouse populations of cabbage looper, *Trichoplusia ni*.. Proc Roy Soc London B.

[14] Tabashnik BE, Gassmann AJ, Crowder DW, Carriere Y (2008). Insect resistance to Bt crops: evidence versus theory.. Nat Biotechnol.

[15] Kruger MJ, Van Rensburg JB, Van den Berg J (2009). Perspective on the development of stem borer resistance to Bt maize and refuge compliance at the Vaalharts irrigation scheme in South Africa.. Crop Prot.

[16] Storer NP, Babcock JM, Schlenz M, Meade T, Thompson GD (2010). Discovery and characterization of field resistance to Bt maize: *Spodoptera frugiperda* (Lepidoptera: Noctuidae) in Puerto Rico.. J Econ Entomol.

[17] Dhurua S, Gujar GT (2011). Field-evolved resistance to Bt toxin Cry1Ac in the pink bollworm, *Pectinophora gossypiella* (Saunders) (Lepidoptera: Gelechiidae), from India.. Pest Manag Sci.

[18] Gassmann AJ, Petzold-Maxwell JL, Keweshan RS, Dunbar MW (2011). Field-evolved resistance to Bt maize by western corn rootworm.. PLoS ONE.

[19] Zhang H, Yin W, Zhao J, Yang Y, Wu S, Tabashnik BE, Wu Y (2011). Early warning of cotton bollworm resistance associated with intensive planting of Bt cotton in China.. PLoS ONE.

[20] US Environmental Protection Agency (1998). Biopesticides registration action document: *Bacillus thuringiensis* plant-incorporated protectants.. http://www.epa.gov/oppbppd1/biopesticides/pips/bt_brad.htm.

[21] Tabashnik BE (2008). Delaying insect resistance to transgenic crops.. Proc Natl Acad Sci U S A.

[22] Tabashnik BE, Dennehy TJ, Carrière Y (2005). Delayed resistance to transgenic cotton in pink bollworm.. Proc Natl Acad Sci U S A.

[23] Henneberry TJ, Naranjo SE (1998). Integrated management approaches for pink bollworm in the southwestern United States.. Integrated Pest Management Rev.

[24] Stone GD (2004). Biotechnology and the political ecology of information in India.. Human Organization.

[25] Carrière Y, Ellers-Kirk C, Kumar K, Heuberger S, Whitlow M (2005). Long-term evaluation of compliance with refuge requirements for Bt cotton.. Pest Manag Sci.

[26] Downes S, Mahon RJ, Rossiter L, Kauter G, Leven T (2010). Adaptive management of pest resistance by *Helicoverpa* species (Noctuidae) in Australia to the Cry2Ab Bt toxin in Bollgard II® cotton.. Evol Appl.

[27] Wu KM, Guo YY (2005). The evolution of cotton pest management practices in China.. Annu Rev Entomol.

[28] Wu KM (2007). Monitoring and management strategy for *Helicoverpa armigera* resistance to Bt cotton in China.. J Invertebr Pathol.

[29] Wu KM, Guo YY, Gao SS (2002). Evaluation of the natural refuge function for *Helicoverpa armigera* (Hübner) within Bt transgenic cotton growing areas in north China.. J Econ Entomol.

[30] Li G, Wu K, Gould F, Wang J, Miao J (2007). Increasing tolerance to Cry1Ac cotton from cotton bollworm, *Helicoverpa armigera*, was confirmed in Bt cotton farming area of China.. Ecol Entomol.

[31] Liu F, Xu Z, Zhu YC, Huang F, Wang Y (2010). Evidence of field-evolved resistance to Cry1Ac-expressing Bt cotton in *Helicoverpa armigera* (Lepidoptera: Noctuidae) in northern China.. Pest Manag Sci.

[32] Ministry of Agriculture (2000). Agricultural Yearbook of China*:* 2000-2010..

[33] Wan P, Wu K, Huang M, Wu J (2004). Seasonal pattern of infestation by pink bollworm *Pectinophora gossypiella* (Saunders) in field plots of Bt transgenic cotton in the Yangtze River Valley of China.. Crop Prot.

[34] Wan P, Zhang Y, Wu K, Huang M (2005). Seasonal expression profiles of insecticidal protein and control efficacy against *Helicoverpa armigera* for Bt cotton in the Yangtze River Valley of China.. J Econ Entomol.

[35] Bambawale OM, Singh A, Sharma OP, Bhosle BB, Lavekar RC (2004). Performance of Bt cotton (MECH-162) under integrated pest management in farmers' participatory field trial in Nanded district, central India.. Current Science.

[36] Bambawale OM, Tanwar PK, Sharma OP, Bhosle BB, Lavekar RC (2010). Impact of refugia and integrated pest management on the performance of transgenic (*Bacillus thuringiensis*) cotton (*Gossypium hirsutum*).. Indian J Agri Sci.

[37] Tabashnik BE, Patin AL, Dennehy TJ, Liu YB, Carrière Y (2000). Frequency of resistance to *Bacillus thuringiensis* in field populations of pink bollworm.. Proc Natl Acad Sci USA.

[38] Tabashnik BE, Fabrick JA, Henderson S, Biggs RW, Yafuso CM (2006). DNA screening reveals pink bollworm resistance to Bt cotton remains rare after a decade of exposure. J Econ Entomol.

[39] Monsanto (2010). Cotton in India.. http://www.monsanto.com/monsanto_today/for_the_record/india_pink_bollworm.asp.

[40] Tabashnik BE, Dennehy TJ, Sims MA, Larkin K, Head GP (2002). Control of resistant pink bollworm (*Pectinophora gossypiella*) by transgenic cotton that produces *Bacillus thuringiensis* toxin Cry2Ab.. Appl Environ Microbiol.

[41] Zhao JZ, Cao J, Li YX, Collins HL, Roush RT (2003). Transgenic plants expressing two *Bacillus thuringiensis* toxins delay insect resistance evolution.. Nat Biotechnol.

[42] Tabashnik BE, Liu YB, Dennehy TJ, Sims MA, Sisterson MS (2002). Inheritance of resistance to Bt toxin Cry1Ac in a field-derived strain of pink bollworm (Lepidoptera: Gelechiidae).. J Econ Entomol.

[43] Liu YB, Tabashnik BE, Dennehy TJ, Patin AL, Sims MA (2001). Effect of Bt cotton and Cry1Ac toxin on survival and development of pink bollworm (Lepidoptera: Gelechiidae).. J Econ Entomol.

[44] Russell RM, Robertson JL, Savin NE (1977). POLO: a new computer program for probit analysis.. Rev Entomol Soc Am.

[45] Tabashnik BE, Biggs RW, Higginson DM, Henderson S, Unnithan DC (2005). Association between resistance to Bt cotton and cadherin genotype in pink bollworm.. J Econ Entomol.

