# Supplementary material for: Increased Frequency of Pink Bollworm Resistance to Bt Toxin Cry1Ac in China
Source: PLoS One. 2012 Jan 4;7(1):e29975. doi: 10.1371/journal.pone.0029975 (PMC3251611; doi:10.1371/journal.pone.0029975)
Supplement: Table S1 — Planting of Bt cotton and non-Bt cotton in the Yangtze River Valley from 2000 to 2010. (DOC) [file pone.0029975.s001.doc]

**Increased Frequency of Pink Bollworm Resistance to Bt Toxin Cry1Ac in China**

Peng Wan^1,2^, Yunxin Huang^3^, Huaiheng Wu^2^, Minsong Huang^2^, Shengbo Cong^2^ , Bruce E. Tabashnik^4^, Kongming Wu^1*^

^1^State Key Laboratory for Biology of Plant Diseases and Insect Pests, Institute of Plant Protection, Chinese Academy of Agricultural Sciences, Beijing, 100193, P.R. China. ^2^Institute of Plant Protection and Soil Science, Hubei Academy of Agricultural Sciences, Wuhan, 430064, P.R. China. ^3^College of Resource and Environment, Hubei University, Wuhan, 430062, P.R. China. ^4^Department of Entomology, University of Arizona, Tucson, Arizona, USA.

**Table S1. Planting of Bt cotton and non-Bt cotton in the Yangtze River Valley from 2000 to 2010.**

| Province | Cotton type |  | Planting area (1,000 ha) | | | | | | | | |  |
| --- | --- | --- | --- | --- | --- | --- | --- | --- | --- | --- | --- | --- |
|  |  | 2000 | 2001 | 2002 | 2003 | 2004 | 2005 | 2006 | 2007 | 2008 | 2009 | 2010 |
|  |  |  |  |  |  |  |  |  |  |  |  |  |
| Sichuan | Bt | 7 | 13 | 20 | 13 | 34 | 24 | 21 | 28 | 36 | 16 | 16 |
|  | Non-Bt | 65 | 53 | 37 | 19 | 15 | 4 | 4 | 3 | 1 | 1 | 1 |
|  |  |  |  |  |  |  |  |  |  |  |  |  |
| Hubei | Bt | 12 | 69 | 68 | 89 | 122 | 195 | 323 | 369 | 408 | 345 | 448 |
|  | Non-Bt | 306 | 278 | 225 | 266 | 286 | 195 | 81 | 50 | 39 | 36 | 27 |
|  |  |  |  |  |  |  |  |  |  |  |  |  |
| Hunan | Bt | 6 | 15 | 17 | 53 | 60 | 77 | 122 | 133 | 148 | 153 | 173 |
|  | Non-Bt | 140 | 135 | 113 | 86 | 108 | 74 | 9 | 13 | 17 | 8 | 5 |
|  |  |  |  |  |  |  |  |  |  |  |  |  |
| Jiangxi | Bt | 1 | 7 | 8 | 26 | 38 | 42 | 53 | 75 | 67 | 77 | 76 |
|  | Non-Bt | 68 | 64 | 47 | 39 | 25 | 22 | 13 | 7 | 7 | 3 | 1 |
|  |  |  |  |  |  |  |  |  |  |  |  |  |
| Anhui | Bt | 62 | 165 | 180 | 254 | 279 | 282 | 334 | 320 | 338 | 333 | 282 |
|  | Non-Bt | 246 | 198 | 141 | 137 | 120 | 94 | 59 | 56 | 37 | 11 | 15 |
|  |  |  |  |  |  |  |  |  |  |  |  |  |
| Jiangsu | Bt | 21 | 63 | 80 | 185 | 246 | 239 | 302 | 301 | 295 | 218 | 211 |
|  | Non-Bt | 274 | 321 | 232 | 185 | 164 | 129 | 53 | 26 | 16 | 12 | 25 |
|  |  |  |  |  |  |  |  |  |  |  |  |  |
| Total | Bt | 109 | 332 | 373 | 619 | 780 | 859 | 1155 | 1225 | 1292 | 1142 | 1205 |
|  | Non-Bt | 1100 | 1049 | 795 | 731 | 718 | 518 | 218 | 155 | 116 | 70 | 74 |
|  | Bt (%) | 9 | 24 | 31.9 | 45.9 | 52.1 | 62.4 | 84.1 | 88.8 | 91.8 | 94.2 | 94.2 |
